# Two cases of linear cutaneous depressions after botulinum toxin A

**DOI:** 10.1016/j.jdcr.2021.08.040

**Published:** 2022-01-19

**Authors:** Timothy Nyckowski, Zackary Whitney, Panagiotis Mitropoulos

**Affiliations:** Department of Dermatology, Kansas City University Graduate Medical Education Consortium, Advanced Dermatology and Cosmetic Surgery, Orlando, Florida

**Keywords:** Abobotulinumtoxin A, atrophy, botox, Botulinum toxin, cosmetic, cutaneous depressions, frontalis, glabella, injection, linear, morphea, morphea-like, neuromodulator, Onabotulinumtoxin A

*To the Editor:* We were pleased to read Landau et al’s^1^ report of morphea-like lesions after botulinum toxin A (BTX A) injections. We additionally report and describe 2 similar cases of this adverse reaction to botulinum toxin while noting a self-resolving nature of these lesions mirroring the average time course for BTX A resolution. The existing literature describes, in total, 5 patients with similar lesions involving the frontalis muscle.^1^, ^2^, ^3^, ^4^ These lesions have been described as “atrophic” or “morphea-like.” Previous hypotheses for this phenomenon include focal muscle atrophy due to a temporary lack of neural stimulation and the contamination of silicone oil syringe lubricant into the skin.^1^^,^^3^

## Case 1

A healthy 26-year-old woman with no medical history and 2 prior episodes of BTX A injections at another facility presented to our office with concerns of lesions on her side of the forehead that appeared after the injection of onabotulinumtoxin A by an outside physician. Three weeks prior, she had received injections in her frontalis and glabellar muscles to treat rhytids. Within days, she noticed discolored skin depressions along the side of the forehead at the injection sites. She returned to her physician for consultation, but the physician could not alleviate the depressions despite 2 follow-up office visits. She then came to our office for further management.

**Fig 1:**
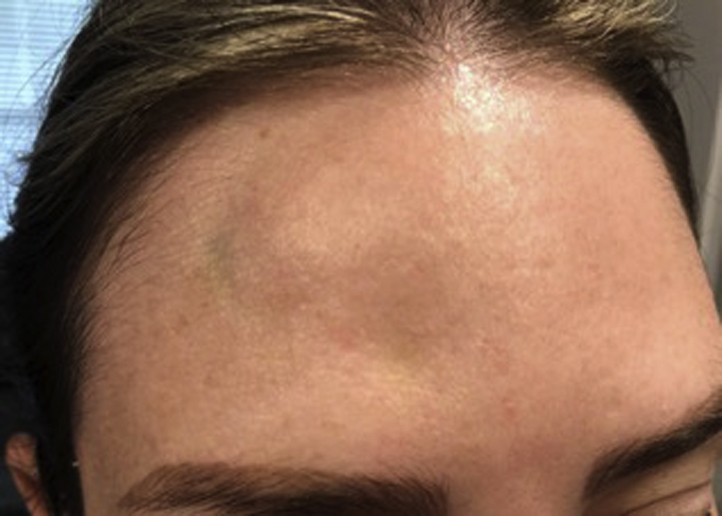
Linear cutaneous depression on the right side of the forehead, starting just below the hairline to approximately the middle aspect of the forehead and extending medially. Bluish-green discoloration noted on the right superior side of the forehead.

**Fig 2:**
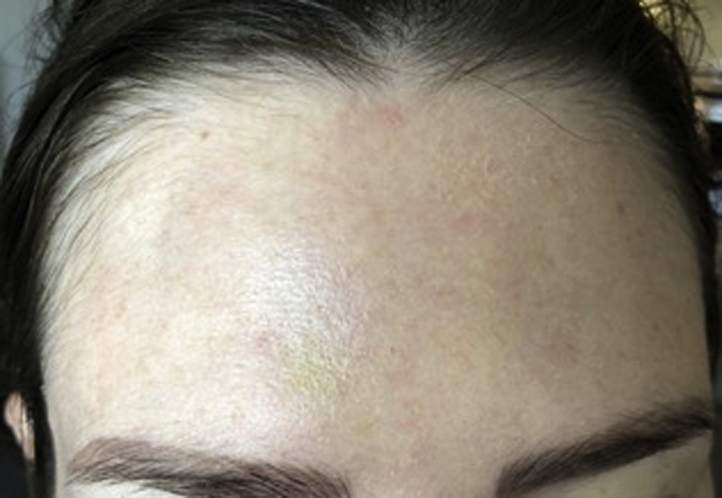
Complete resolution of the cutaneous depressions after 3 months. Some minimal discoloration still present superior to the medial aspect of the right brow.

On physical examination, an atrophic plaque with an underlying blue-green color change was noted (Fig 1). The injecting physician confirmed that the standard dilution was used for onabotulinumtoxin A and confirmed that no other substance was used. The patient was scheduled for normal saline injections to the atrophic areas every 2 to 4 weeks, which resulted in temporary improvement. After 3 months’ time, these lesions resolved without other intervention (Fig 2). Since the time of this adverse event, she has received an additional BTX A treatment for rhytids with our office, without the recurrence of these cutaneous depressions.

### Case 2

A 27-year-old woman with no medical history presented to our office with lesions on the central aspect of the forehead that appeared after the glabellar and frontalis injection of abobotulinumtoxin A by a physician assistant 4 weeks prior. Within days after the initial injection, she noticed soft-tissue swelling in the central aspect of the forehead area. Her injector was unsure how to proceed with the treatment, so she came to our office for further management.

**Fig 3:**
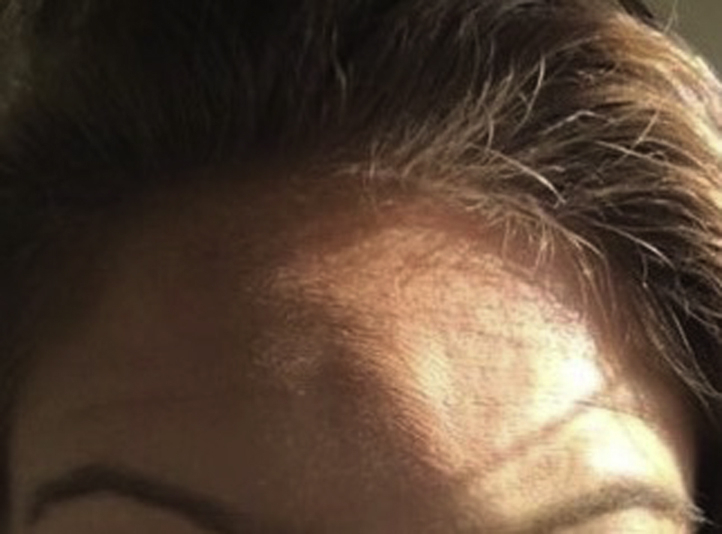
Central linear depression, with mild swelling flanking the medial aspect of the forehead area bilaterally.

**Fig 4:**
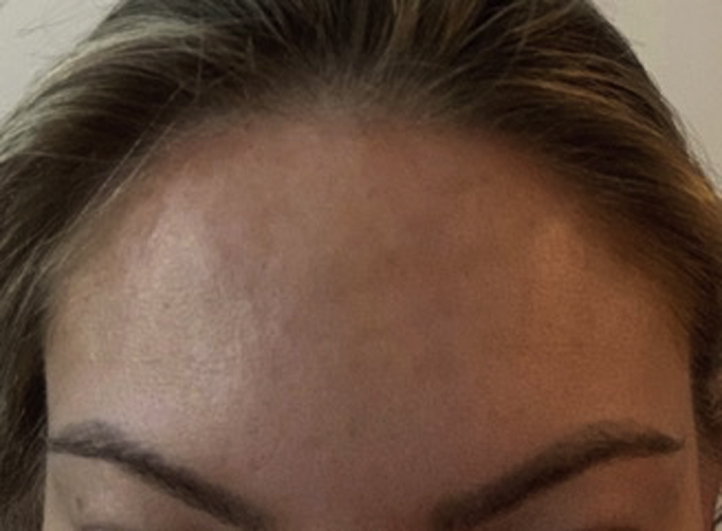
Resolution of the cutaneous linear depression in the central aspect of the forehead. No swelling or discoloration present.

On physical examination, there was a large central linear cutaneous depression, best appreciated on tangential lighting (Fig 3). We administered normal saline injections to the affected area weekly for 4 months. Biopsy was considered, but, ultimately, the lesions gradually improved until disappearance, 3.5 months after the initial appearance (Fig 4).

Both of our patients had resolution of the cutaneous depressions within 1 month of the standard duration of BTX A, which is around 3 to 6 months.^5^ This is consistent with most of the previously described patients, with the exception of a case described in the literature of a patient who had received 185 total units of BTX A injections for headaches—far greater than what we typically use to treat facial rhytids. Yet, even in this circumstance, the patient had almost complete resolution of the depressions within 6 months and entirely within 12 months.

Given the similar duration of BTX A and the cutaneous depressions, it seems as though aberrant muscle contraction may be the underlying mechanism for this adverse effect. Since the frontalis muscle has cutaneous insertions, changes in the contractility of its fibers may lead to superficial skin changes.^6^ Specifically, the area of depressed skin may have increased muscle contraction relative to the surrounding skin. The increased contraction may be due to complex neuromuscular mechanisms involving the retrograde axonal transport of the BTX A, ultimately leading to less inhibitory input to the muscle fibers, causing cutaneous depression.^7^^,^^8^ Alternatively, the BTX A may act on the perilesional musculature, leaving unaffected musculature in a state of relative hypertonicity.

There are 2 variants of cutaneous depressions after BTX A injection: 1 with well-defined circular depressions and 1 that appears more linear, following the orientation of the frontalis muscle. Linear depressions, such as ours, are more likely due to aberrant frontalis muscle contraction, given the underlying anatomy.^6^ Round depressions may be due to direct atrophy, evidenced by the action halo of the neuroprotein, as previously hypothesized.^3^ Silicone oil contamination may also contribute to the cutaneous depressions, but further studies are warranted.

Linear cutaneous depression after BTX A injection was demonstrated in 1 previous report, in which the patient was biopsied for concern of coup de sabre, but this biopsy showed no abnormalities.^3^ We would like to reassure injectors that a biopsy for linear depressions after BTX A injections may be deferred until 3 to 6 months after injection, during the time when the BTX A effect should resolve. Additionally, serial normal saline injections may temporarily improve the appearance. Further studies and reports are encouraged to confirm our findings.

## Conflicts of interest

None disclosed.
